# Long-term quality of life and sexual function of elderly people with endometrial or ovarian cancer

**DOI:** 10.1186/s12955-021-01675-2

**Published:** 2021-02-12

**Authors:** Ariane Mamguem Kamga, Leila Bengrine-Lefevre, Valérie Quipourt, Laure Favier, Ariane Darut-Jouve, Sophie Marilier, Patrick Arveux, Isabelle Desmoulins, Tienhan Sandrine Dabakuyo-Yonli

**Affiliations:** 1 Epidemiology and Quality of Life Research Unit, Lipids, Nutrition, Cancer Research Center, INSERM U1231, Georges Francois Leclerc Centre – UNICANCER, 1 rue Professeur Marion, BP 77980, 21079 Dijon Cedex, France; 2 Medical Oncology Department, Centre Georges-François Leclerc, 1 rue Pr. Marion, 21000 Dijon, France; 3 Geriatric Oncology Coordination Unit in Burgundy, University Hospital, 21079 Dijon, France; 4 Department of Geriatrics and Internal Medicine, Hospital of Champmaillot, University Hospital, 21079 Dijon, France; 5 Oncology Centre of Park, 18 Cours du General De Gaulle, Dijon, France; 6 Centre for Research in Epidemiology and Population Health (CESP), INSERM U1018, University Paris-Sud, UVSQ Gustave Roussy, Villejuif, France; 7 National Quality of Life and Cancer Platform, Dijon, France

**Keywords:** Health-related quality of life, Endometrial cancer, Ovarian cancer, Old patients

## Abstract

**Background:**

With the growing number of older endometrial cancer (EC) and ovarian cancer (OC) survivors, data on long-term health-related quality of life (HRQoL) became an important issue in the management of older patients. So, the aim of this study was to describe and compare according to age long-term HRQoL, sexual function, and social deprivation of adults with either EC or OC.

**Methods:**

A cross-sectional study was set up using data from the Côte d’Or gynecological cancer registry. A series of questionnaires assessing HRQoL (SF-12), sexual function (FSFI), anxiety/depression (HADS), social support (SSQ6) and deprivation (EPICES) were offered to women with EC or OC diagnosed between 2006 and 2013. HRQoL, sexual function, anxiety/depression, social support and deprivation scores were generated and compared according to age (< 70 years and ≥ 70 years).

**Results:**

A total of 145 women with EC (N = 103) and OC (N = 42) participated in this study. Fifty-six percent and 38% of EC and OC survivors respectively were aged 70 and over. Treatment did not differ according to age either in OC or EC. The deprivation level did not differ between older and younger survivors with OC while older survivors with EC were more precarious. The physical HRQoL was more altered in older EC survivors. This deterioration concerned only physical functioning (MD = 24, *p* = 0.012) for OC survivors while it concerned physical functioning (MD = 30, *p* < 0.0001), role physical (MD = 22, *p* = 0.001) and bodily pain (MD = 21, *p* = 0.001) for EC survivors. Global health (MD = 11, *p* = 0.011) and role emotional (MD = 12, *p* = 0.018) were also deteriorated in elderly EC survivors. Sexual function was deteriorated regardless of age and cancer location with a more pronounced deterioration in elderly EC survivors for desire (*p* = 0.005), arousal (*p* = 0.015) and orgasm (*p* = 0.007). Social support, anxiety and depression were not affected by age regardless of location.

**Conclusion:**

An average 6 years after diagnosis, the impact of cancer on HRQoL is greatest in elderly survivors with either EC or OC.

## Background

Endometrial and Ovarian cancers are primarily a disease of older people with median age at diagnosis respectively of 69 and 68 years old [1]. With population living longer, the number and proportion of older adults will increase, along with the incidence of both endometrial and ovarian cancer [2, 3]. Elderly women with endometrial cancer have histologically aggressive tumors and are frequently diagnosed at advanced stages of the disease [4, 5]. Ovarian cancer (OC) in elderly women is mainly diagnosed at a higher stage [6, 7]. Managing cancer in older people is very complex. Older people with cancer generally are likely to have several comorbidities with polypharmacy as consequences and physiologic age‐related changes [8]. Surgical treatment completed by adjuvant radiotherapy and systemic therapy depending on the stage of disease is the standard treatment for endometrial cancer [9]. For OC, treatment include surgical staging and cytoreduction as well as systemic chemotherapy, either as adjuvant treatment or neoadjuvant therapy before surgery [10]. Despite recommendations, older women with endometrial or ovarian cancer are undertreated [4, 11] and underrepresented in clinical trials [12]. Outcomes in older women with OC is known to be worse. Indeed, older patients with OC show an impaired survival prognosis compared to younger patients [2, 11]. For elderly patients with endometrial cancer (EC), cancer specific survival rate was lower among the elderly and very elderly women than among their younger counterparts [5, 13, 14]. With the growing number of older endometrial and ovarian cancer survivors, data on long-term health-related quality of life (HRQoL) became an important issue in the management of older patients. Few studies has focused on this issue in regard with long term. So, the aim of this study was to describe and compare according to age the long-term HRQoL, sexual function, and social deprivation of people with endometrial or ovarian cancer.

## Methods

This study is an ancillary study. The primary study aimed to identify determinants of long term HRQoL in women with cervical, endometrial and ovarian cancer and to describe their living conditions. The main study was performed in accordance with the Declaration of Helsinki and have received ethics approval. Indeed, the study was approved by the french national data protection authority (Commission nationale de l’informatique et des libertés MR003 N° 1989764 V.0). Also, women have received legal information about the study. The response to the questionnaires constituted the women's consent to participate. Details regarding the study design, sample, and methods have been previously reported [15]. For this study, eligible participants were required to be women aged 18 years old and more; to be diagnosed with endometrial or ovarian cancer between 2006 and 2013; to be identified through Côte d'Or breast and gynecological cancer registry and to be alive on the 1st January 2017. The Côte d'Or breast and gynecological cancer registry is the only one in France to focus on breast and gynecologic cancers. It has been collecting data on all cases of breast and gynecologic cancer occurring in residents of Côte d’Or since 1982.

Concerning data collection, clinical characteristics like age at diagnosis, stage, treatments, comorbidities, hormonal status were extracted from the breast and gynecological cancers registry of Côte d’Or.

Age at the time of survey was also collected. In January 2017, women eligible received a mail including an information letter and some questionnaires. These questionnaires included the Medical Short-Form 12 (SF-12) [16] to assess HRQoL, the Female Sexual Function Index (FSFI) [17, 18] for sexual function, the hospital anxiety and depression scale (HADS) [19] for anxiety/depression, the Sarason social support questionnaire (SSQ6) [20] for social support and the EPICES (Évaluation de la précarité et des inégalités de santé pour les centres d’examen de santé) questionnaire [21] for individual deprivation. In the absence of any response from patients within 1 month, a reminder was sent.

The Medical Outcomes Study 12-item Short Form health survey (SF-12) is a validated tool used to assess general HRQoL [16]. It comprises eight scales and two summary scales, namely the Physical Component Summary (PCS) and the Mental Component Summary (MCS) can be computed from the eight scales.

The Female Sexual Function Index (FSFI) is a self-reported measurement of sexual functioning developed by Rosen [17]. A French version has been validated in women including those with cancer [18]. This 19-item questionnaire explores sexual function in six dimensions: desire, arousal, lubrication, orgasm, sexual satisfaction, and pain of intercourse.

The Hospital Anxiety and Depression Scale (HADS) is an instrument for detecting anxiety and depressive disorders. It was validated and adapted in French in 1989 by Lepine et al. [19]. This scale has 14 items rated from 0 to 3 and covers 2 dimensions.

Sarason’s six-item Social Support questionnaire (SSQ6) is a validated tool adapted in French by Rascle et al. [20]. This questionnaire reflects the support available in patients’ environment. Social support is measured across 2 dimensions: support availability, through the number of contacts that the patient can count on (0 to 9 people) and quality of support, through patient satisfaction with support received.

The EPICES questionnaire [21] is a questionnaire developed specifically for the French context that contains 11 items that take into account the overall living conditions. It explores individual deprivation and social health and discriminates the most deprived (score > 30) from non-deprived individuals (score ≤ 30).

For each location, we compared respondents and non-respondents on their clinical and personal characteristics. Clinical and personal characteristics were also compared according to age for the two locations. We performed univariate analyses to compare HRQOL, sexual function, anxiety/depression, social support and individual deprivation scores for each location and according to age at the time of the survey (< 70 years and ≥ 70 years). A *p* value lower than 0.05 was set to define a statistically significant difference. We also performed a mixed regression model on clinically meaningful HRQoL dimensions to found if age was a significant determinant of HRQoL adjusted on other variables. We define clinically meaningful HRQoL dimensions as dimensions in which the mean difference between older and younger were more than 10 points. The eligibility threshold for the multivariable model was a *p* value less than 0.20.

## Results

### Characteristics

Three hundred and fourteen women was eligible and 145 (46%) women participated to the study. Among them, we have respectively 103 and 42 women with EC and OC. There is a significant difference between responders and non-responders for EC in terms of time since diagnosis (Table 1). Women with a longer time since diagnosis were reluctant to participate to the study (*p* = 0.01).

**Table 1:** Comparison between responders and non-responders in ovarian and endometrial cancer survivors

Variables	Ovarian Cancer Survivors					Endometrial cancer survivors				
	Responders		Non-responders		*p*	Responders		Non-responders		*p*
	N = 42	%	N = 27	%		N = 103	%	N = 142	%	
*Time since diagnosis (m)*					0.79					0.06
Mean (SD)	76.1 (27.1)		77.8 (26.5)			80.2 (29.3)		87.2 (26.3)		
Median [range]	71.0 [39.0–127.0]		73.0 [37.0–127.0]			76.0 [36.0–131.0]		90.5 [37.0–130.0]		
*Age at diagnosis (years)*					0.92					0.85
Mean (SD)	59.1 (12.5)		60.1 (13.7)			65.1 (9.4)		65.0 (9.0)		
Median [range]	59.5 [20.0–82.0]		59.0 [30.0–88.0]			65.0 [42.0–86.0]		65.0 [37.0–86.0]		
*Time since diagnosis*					0.6					0.01
< 5 years	15	35.7	8	29.6		34	33.0	27	19.0	
≥ 5 years	27	64.3	19	70.4		69	67.0	115	81.0	
*Stage*					0.75					0.25
I	17	41.5	13	48.1		94	92.2	121	85.2	
II	5	12.2	4	14.8		3	2.9	9	6.3	
III	19	46.3	10	37.1		5	4.9	12	8.4	
Unknown	1		0			1		0		
*Hormonal status*					0.56					> 0.99
Postmenopausal	35	83.3	21	77.8		100	97.1	137	96.5	
Not postmenopausal	7	16.7	6	22.2		3	2.9	5	3.5	
*CCI*					0.43					> 0.99
0	23	88.5	15	79.0		60	66.7	84	66.7	
≥ 1	3	11.5	4	21.0		30	33.3	42	33.3	
Unknown	16		8			13		16		
*Surgery*					0.39					0.41
Yes	42	100.0	26	96.3		100	99.1	142	100.0	
No	0	0.0	1	3.7		1	0.9	0	0.0	
Unknown						2				
*Chemotherapy*					0.51					0.50
Yes	31	73.8	21	80.8		5	4.9	10	7.0	
No	11	26.2	5	19.2		96	95.1	132	93.0	
Unknown	0		1			2		0		
*Radiotherapy*					0.39					0.06
Yes	0	0.0	1	3.7		67	66.3	108	77.1	
No	42	100.0	26	96.3		34	33.7	32	22.9	
Unknown						2		2		
*Treatments*					0.77					0.14
Surgery alone	11	26.2	6	23.1		31	31.0	32	22.5	
Surgery ± radiotherapy ± chemotherapy	31	73.8	20	76.9		69	69.0	110	77.5	
Unknown	0		1			3		0		

*SD* standard deviation, *CCI* Charlson Comorbidity Index

Fifty-six percent and 38% of endometrial and ovarian cancer survivors were aged 70 and over respectively by the time of the survey. Treatments did not differ between younger and older survivors in both cancer locations (*p* = 0.48 for EC and *p* = 0.72 for OC). While OC survivors were treated mostly with chemotherapy, EC survivors on their own were treated with radiotherapy. The level of individual deprivation did not differ between older and younger OC survivors, while older survivors with EC were more precarious. The complete description of endometrial and ovarian cancer survivors are displayed in Table 2.

**Table 2:** Clinical and sociodemographic characteristics by age for ovarian and endometrial cancer survivors

Variables	Ovarian cancer survivors					Endometrial cancer survivors				
	< 70 years		≥ 70 years		*p* value	< 70 years		≥ 70 years		*p* value
	N = 26	%	N = 16	%		N = 45	%	N = 58	%	
*Time since diagnosis (months)*					0.16					0.43
Mean (SD)	71.8 (24.1)		84.7 (30.4)			77.5 (27.8)		82.3 (30.4)		
Median [range]	69.0 [39.0–122.0]		80.5 [46.0–127.0]			74.0 [36.0–130.0]		79.0 [37.0–131.0]		
*Age at diagnosis (years)*					< 0.001					< 0.001
Mean (SD)	52.2 (10.6)		70.2 (5.1)			56.9 (5.0)		71.4 (6.9)		
Median [range]	56.0 [20.0–65.0]		70.5 [63.0–82.]			58.0 [42.0–66.0]		72.0 [60.0–86.0]		
*Time since diagnosis*					0.85					0.72
< 5 Years	9	34.6	6	37.5		14	31.1	20	34.5	
≥ 5 Years	17	65.4	10	62.5		31	68.9	38	65.5	
*Stage*					0.90					0.04
I	11	44.0	6	37.5		44	100.0	50	86.2	
II	3	12.0	2	12.5		0	0.0	3	5.2	
III	11	44.0	8	50.0		0	0.0	5	8.6	
Unknown	1		0			1		0		
*CCI*					> 0.99					0.13
0	13	86.7	10	90.9		30	75.0	30	60.0	
≥ 1	2	13.3	1	9.1		10	25.0	20	40.0	
Unknown	11		5			5		8		
*BMI*					0.52					0.13
≤ 25	14	53.8	7	43.7		24	55.6	40	70.2	
> 25	12	46.2	9	56.3		20	44.4	17	29.8	
Unknown	0		0			1		1		
*Hormonal status*					0.03					0.31
Postmenopausal	19	73.1	16	100.0		42	93.3	58	100.0	
Not postmenopausal	7	26.9	0	0.0		3	6.7	0	0.0	
*Surgery*										> 0.99
Yes	26	100.0	16	100.0		44	100.0	56	98.3	
No	0	0.0	0	0.0		0	0.0	1	1.7	
Unknown	0		0			1		1		
*Chemotherapy*					0.72					0.65
Yes	20	76.9	11	68.7		3	6.8	2	3.5	
No	6	23.1	5	31.3		41	93.2	55	96.5	
Unknown	0		0			1		1		
*Radiotherapy*										0.73
Yes	0	0.0	0	0.0		30	68.2	37	35.1	
No	26	100.0	16	100.0		14	31.8	20	64.9	
Unknown	0					1		1		
*Treatments*					0.72					0.48
Surgery alone	6	23.1	5	31.3		13	29.6	18	32.1	
Surgery ± radiotherapy ± chemotherapy	20	76.9	11	68.7		31	70.4	38	67.9	
Unknown	0		0			1		2		
*EPICES score*					0.70					< 0.001
EPICES ≤ 30	19	79.2	10	71.4		38	92.7	22	53.7	
EPICES > 30	5	20.8	4	28.6		3	7.3	19	46.3	
Unknown	2		2			4		17		

*SD* standard deviation, *CCI* Charlson Comorbidity Index, *BMI* Body Mass Index

### HRQoL, sexual function, social support, anxiety and depression

Among ovarian cancers survivors, HRQoL was not good. The mean physical component score was 49 (± 7.5) for younger ones and 40.8 (± 10.5) for older ones. Physical functioning (MD = 24, *p* = 0.01) was the only dimension statistically and clinically meaningful among OC survivors; bodily pain (MD = 14, *p* = 0.1) and role physical (MD = 15, *p* = 0.08) were only clinically meaningful. Sexual function was impaired regardless of age. See Table 3 for more details.

**Table 3:** HRQoL and sexual function scores by age among ovarian cancer survivors

Variables	< 70 years			≥ 70 years			*p* value
	N = 26	Mean (SD)	Median (range)	N = 16	Mean (SD)	Median (range)	
*HRQoL*							
General health	26	57.3 (21.2)	60.0 (25.0–100.0)	16	50.6 (18.9)	60.0 (25.0–85.0)	0.30
Physical functioning	25	84.0 (26.9)	100.0 (0.0–100.0)	16	59.4 (36.4)	75.0 (0.0–100.0)	0.01
Role physical	26	67.8 (21.3)	62.5 (37.5–100.0)	16	52.3 (28.9)	50.0 (0.0–100.0)	0.08
Role emotional	26	61.1 (25.1)	50.0 (0.0–100.0)	16	56.2 (27.4)	50.0 (0.0–100.0)	0.49
Bodily pain	26	70.2 (27.4)	75.0 (0.0–100.0)	16	56.2 (26.6)	50.0 (0.0–100.0)	0.10
Mental health	26	59.6 (24.3)	62.5 (0.0–100.0)	16	63.3 (19.6)	62.5 (37.5–100.0)	0.79
Vitality	25	52 (21.5)	50.0 (0.0–100.0)	16	45.3 (30.6)	50.0 (0.0–100.0)	0.32
Social functioning	26	64.4 (26.6)	75.0 (0.0–100.0)	16	64.1 (27.3)	62.5 (25.0–100.0)	0.89
PCS	24	49 (7.5)	48.2 (36.9–66.2)	16	40.8 (10.5)	42.9 (17.5–55.2)	0.02
MCS	24	41.6 (10.5)	40.4 (17.5–60.3)	16	43.6 (10.0)	43.3 (30.1–66.0)	0.69
*Sexual function*							
Desire	23	2.2 (1.2)	1.8 (1.2–5.4)	13	2 (1.3)	1.2 (1.2–4.8)	0.41
Arousal	24	1.8 (2.2)	0.6 (0.0–6.0)	14	1.4 (1.7)	0.6 (0.0–4.8)	0.67
Pain	21	1.9 (2.5)	0.0 (0.0–6.0)	11	0.7 (1.6)	0.0 (0.0–4.8)	0.15
Satisfaction	15	3.3 (2.0)	3.2 (0.8–6.0)	5	3.8 (2.0)	4.4 (0.8–6.0)	0.63
Lubrication	23	1.5 (2.2)	0.0 (0.0–6.0)	13	0.8 (1.8)	0.0 (0.0–6.0)	0.68
Orgasm	24	1.7 (2.3)	0.0 (0.0–6.0)	13	0.8 (1.6)	0.0 (0.0–4.4)	0.23
Global Score	15	15.4 (12.3)	22.1 (2.0–35.4)	4	16.3 (9.6)	18.7 (3.5–24.2)	0.88

*HRQoL* Health-Related Quality of Life, *PCS* Physical Component Score, *MCS* Mental Component Score, *SD* standard deviation

Among older survivors with EC, the physical component of HRQoL was more degraded compared to younger ones. Indeed, EC survivors aged less than 70 years old have a mean physical component score of 48.2 (± 10.6) while the older ones have a score of 37.8 (± 9.9). Physical functioning (MD = 30, *p* < 0.001), role physical (MD = 22, *p* = 0.001) and bodily pain (MD = 21, *p* = 0.001), general health (MD = 11, *p* = 0.01) and role emotional (MD = 12, *p* = 0.02) were the dimensions statistically and clinically meaningful among EC survivors. Sexual function was impaired regardless of age with greater deterioration in EC survivors aged 70 and over for the dimensions desire (*p* = 0.01), arousal (*p* = 0.01) and orgasm (*p* = 0.01). The complete description of HRQoL and sexual function could be found in Table 4.

**Table 4:** HRQoL and sexual function scores by age among endometrial cancer survivors

Variables	< 70 years			≥ 70 years			*p* value
	N = 45	Mean (SD)	Median (range)	N = 58	Mean (SD)	Median (range)	
*HRQoL*							
General health	44	64.4 (22.8)	60.0 (25.0–100.0)	57	52.7 (21.7)	60.0 (0.0–100.0)	0.01
Physical functioning	44	73.9 (32.3)	87.5 (0.0–100.0)	55	43.2 (37.4)	50.0 (0.0–100.0)	< 0.001
Role physical	44	67.6 (30.5)	75.0 (0.0–100.0)	56	45.5 (27.8)	50.0 (0.0–100.0)	0.001
Role emotional	45	65.3 (27.3)	62.5 (0.0–100.0)	53	52.4 (26.4)	50.0 (0.0–100.0)	0.02
Bodily pain	43	75.0 (27.8)	75.0 (0.0–100.0)	55	54.1(26.7)	50.0 (0.0–100.0)	0.001
Mental health	44	60.5 (20.2)	62.5 (12.5–100.0)	56	56.2 (20.8)	50.0 (0.0–100.0)	0.28
Vitality	43	47.1 (27.4)	50.0 (0.0–100.0)	54	43.0 (25.0)	50.0 (0.0–100.0)	0.35
Social functioning	44	72.2 (26.0)	75.0 (0.0–100.0)	56	63.4 (24.7)	75.0 (25.0–100.0)	0.07
PCS	41	48.2 (10.6)	50.3 (20.4–61.5)	51	37.8 (9.9)	38.3 (16.7–60.8)	< 0.001
MCS	41	43.4 (10.9)	44.8 (16.9–61.1)	51	42.2 (9.1)	41.9 (25.6–62.4)	0.38
*Sexual function*							
Desire	43	2.4 (1.2)	2.4 (1.2–6.0)	46	1.7 (0.9)	1.2 (1.2–3.6)	0.01
Arousal	43	2.1 (2.0)	1.8 (0.0–6.0)	42	1.0 (1.3)	0.3 (0.0–4.5)	0.01
Pain	40	1.7 (2.2)	0.0 (0.0–6.0)	42	0.9 (1.8)	0.0 (0.0–6.0)	0.07
Satisfaction	27	3.6 (1.8)	4.0 (0.8–6.0)	18	2.9 (1.5)	3 (0.8–5.2)	0.14
Lubrication	42	1.7 (2.1)	0.6 (0.0–6.0)	42	1.1 (1.8)	0.0 (0.0–6.0)	0.07
Orgasm	43	2.0 (2.3)	1.2 (0.0–6.0)	44	0.8 (1.4)	0.0 (0.0–4.8)	0.01
Global score	26	17.4 (10.5)	19.1 (2.0–36.0)	18	13.5 (8.7)	14.8 (2.0–26.8)	0.20

*HRQoL* Health-Related Quality of Life, *PCS* Physical Component Score, *MCS* Mental Component Score, *SD* standard deviation

Social support, anxiety and depression were not affected by age regardless of location (Table 5).

**Table 5:** Social support, anxiety and depression by age among ovarian endometrial cancer survivors

Variables	< 70 years			≥ 70 years			*p* value
	N	Mean (SD)	Median (range)	N	Mean (SD)	Median (range)	
*Ovarian cancer*	N = 26			N = 16			
*Social support*							
Social support availability	22	15.4 (9.1)	15.0 (0.0–36.0)	11	11.9 (4.6)	11.0 (6.0–22.0)	0.32
Social support satisfaction	18	29.0 (7.1)	30.0 (6.0–36.0)	10	29.2 (4.5)	29.0 (22.0–36.0)	0.56
*HADS scores*							
Anxiety	25	8.8 (4.9)	9.0 (0.0–19.0)	16	8.2 (3.7)	7.5 (3.0–15.0)	0.71
Depression	24	5.0 (2.8)	5.0 (1.0–10.0)	15	6.1 (3.6)	7.0 (0.0–11.0)	0.35
Anxiety (%)							
< 11	15	60.0		10	62.5		
≥ 11	10	40.0		6	37.5		
Missing	1			0			
Depression (%)							0.14
< 11	24	100.0		13	86.7		
≥ 11	0	0.0		2	13.3		
Missing	2			1			
*Endometrial cancer*	N = 45			N = 58			
*Social support*							
Social support availability	39	14.2 (7.5)	12.0 (4.0–31.0)	43	15.6 (10.5)	14.0 (0.0–42.0)	0.86
Social support satisfaction	35	28.7 (5.7)	29.0 (16.0–36.0)	34	29.3 (6.1)	30.0 (6.0–36.0)	0.54
*HADS scores*							
Anxiety	44	9.0 (4.1)	9 (2.0–20.0)	48	8.7 (4.3)	7.0 (0.0–18.0)	0.64
Depression	43	5.8 (4.8)	6 (0.0–20.0)	49	6.6 (3.4)	6.0 (1.0–15.0)	0.11
Anxiety (%)							0.46
< 11	28	63.6		34	70.8		
≥ 11	16	36.4		14	29.2		
Missing	1			10			
Depression (%)							0.65
< 11	39	90.7		43	87.8		
≥ 11	4	9.3		6	12.2		
Missing	2			9			

*SD* standard deviation, *HADS* Hospital Anxiety and Depression Scale

Age was a significant determinant for physical functioning and bodily pain subscales among EC survivors whereas it was significant only for physical functioning among OC survivors when adjusting on others variables (Table 6).

**Table 6:** Significant determinants of health-related quality of life

SF-12 significant dimensions	Variables	Estimate	Standard error	*p* value
*Endometrial cancer*				
Physical functioning^a^	Charlson score at diagnosis			0.0027
	0	0		
	≥ 1	− 18.6824	6.0810	
	Age by the time of survey			< 0.0001
	< 70	25.9086	5.6407	
	≥ 70	0		
Bodily pain^b^	BMI			0.0028
	≤ 25	0		
	> 25	− 14.2069	4.6464	
	Depression			0.0037
	< 11	0		
	≥ 11	− 21.1004	7.1137	
	EPICES deprivation score			0.0049
	≤ 30	0		
	> 30	− 14.8791	5.1845	
	Age by the time of survey			0.0054
	< 70	13.3102	4.6935	
	≥ 70	0		
*Ovarian cancer*				
Physical functioning^c^	Age by the time of survey			0.0035
	< 70	28.8333	9.1607	
	≥ 70	0		

^a^Adjusted on BMI, stage, comorbidity, time since diagnosis, depression, EPICES deprivation score, radiotherapy

^b^Stage, time since diagnosis, comorbidity, chemotherapy, radiotherapy

^c^BMI, anxiety, depression

## Discussion

This study analyzed 103 women with EC and 42 women with OC with an average time since diagnosis of 6 years. As shown in the original study, HRQoL was deteriorated among women with EC and OC. The results of this study indicate that this deterioration was more pronounced in older women with EC. Indeed, older women with EC in this study had a worst score on all subscales except mental health, social functioning and vitality compared to youngers ones.

Age was found as great determinant of physical functioning and bodily pain for EC survivors. For women with OC, age was a significant determinant of HRQoL. Physical functioning was impaired among older survivors compared to younger ones.

With advancing age, many changes occur which affect all the organs system. These changes led to age-related diseases, functional changes capacities and ultimately to organ failures [22]. Older patients with gynecological cancer are not exception. Indeed, they may have aging-associated frailties associated with comorbidities and geriatric syndromes. There was a more pronounced physical impairment among older survivors with EC as opposed to older OC survivors. This could be explained by the impact of treatment but also co-morbidities which are more present in patient with EC. Medical problems such as hypertension, obesity, articular dysfunction can lead to a worse functional status and vitality impairment. EC survivors in this study were treated primarily with radiotherapy. Unlike chemotherapy known for its short-term effects, radiotherapy can have a significant physical impact in the long term [23]. Radiotherapy lead to vaginal dryness conducting to sexual alteration. Vaginal suture conduct to anatomical modification and impact sexual activities. Moreover, compare to younger ones, outcome of older survivors of EC treated by radiotherapy is worse [14].

Concerning older OC survivors in this study, physical impairment was the only problem reported by the older ones compared to younger ones. This result corroborate that of Van Walree et al. [7]. In their study, they had found that physical functioning subscale score of older women with OC is worse than those of younger ones. Comorbidities are less present among those women and cannot explain the difference. This physical impairment may be chemotherapies-related particularly with platin and taxan. Fatigue and neuropathy are the most common side effects of these treatments. Recovery could be slower in older people and conduct in more physical impairment. Nevertheless, we can excluded the possibility of lack of power due the low number of OC survivors.

Sexual function scores were low regardless of age and cancer location with lower scores in EC survivors aged 70 and more. Endometrial and ovarian cancers being part of gynecological cancers are known to be associated with a greater risk of sexual impairment particularly due to their location (genital tract) and treatment which is localized (as surgery and other) [24]. Sexual issues go unaddressed for many cancer survivors, particularly women [25]. There is a great lack of assessment of sexual function during medical monitoring after treatment. Few providers often ask patients about sexual concerns and patients don’t really raised the topic [26]. Women with gynecologic cancers also pointed worries and lack of information to worsen sexual impairment [27]. So, a sexual consultation could be a way to fought sexual dysfunction despite treatment sequelae. Nevertheless, we must be cautious in interpreting these results because of the high level of missing data in sexual dysfunction, particularly for satisfaction items.

Depression is considered as a poor prognostic marker of physical disability in elderly patients with cancer [28] during treatment. In this long term study, depression level is not different between older and younger survivors regardless of location.

Although a low rate response and a low number of patients, this study shows us long-term results. Moreover, the results of the study show that old patients respond to the solicitation. This result is along of that of Prieske et al. [29]. In their study assessing the disposition and apprehension of elderly patients (aged ≥ 65 years old) with gynecological cancers toward study participation had found that old patients are generally willing to participate in clinical studies. Although recommendations have been made concerning the need for older patients to take part in research studies and randomized clinical trials devoted to HRQoL, much remains to be done to ensure that these measures are effective. Nevertheless, efforts have made recently to ensure that this is the case with some studies focusing on older patients being conducted [6, 7, 23].

## Conclusion

In conclusion, this study shows us long-term results of HRQoL and sexual function of old patients with either endometrial or ovarian cancers. Older patients may have aging-associated frailties associated with comorbidities and geriatric syndromes. The more marked physical deterioration in EC survivors could be explained by the impact of treatments (radiotherapy versus chemotherapy) but also comorbidities, not to mention the intrinsic prognosis of cancers.

## Data Availability

Data are available on request from the authors.

## Abbreviations

HRQoL: Health-Related Quality Of Life
EC: Endometrial cancer
OC: Ovarian cancer
MD: Mean difference
SF-12: Medical Short Form 12
FSFI: Female Sexual Function Index
HADS: Hospital Anxiety and Depression Scale
SSQ6: Sarason Social Support Questionnaire
EPICES: Évaluation de la précarité et des inégalités de santé pour les centres d’examen de santé
